# Comparing the linkage performance of fastLink, Match*Pro and Splink at the Florida Cancer Registry using simulated pseudopeople data

**DOI:** 10.23889/ijpds.v9i5.2786

**Published:** 2024-09-18

**Authors:** Anders Alexandersson

**Affiliations:** 1 Florida Cancer Registry; 2 University of Miami

## Objective

The objective was to compare the record linkage performance of fastLink, Match*Pro, and Splink using realistic simulated "Rhode Island" data from the Python package pseudopeople. A requirement was no expected wrong matches (false positives, FP) after a limited manual review.

## Approach

We incorporated custom noise on (a) Social Security Number (SSN) and (b) address. We first used high-quality data where SSN was available and then low-quality data where SSN was unavailable. Missed matches (false negatives, FN) were acceptable. We used an iterative process of determining the “appropriate” match threshold and the correct matches (true positives, TP).

## Results

The two analysis datasets, df1 and df2, resulted in 660,227 actual matches and 9,987 actual non-matches. The specified match threshold probability was 0.995 for fastLink, 0.999999999 for Splink, and about 0.99 for Match*Pro. When SSN was available, the (rounded) results were: Splink 627,000 TP (4 FP), fastLink 577,000 TP (15 FP), and Match*Pro 436,000 TP (4 FP) in the default frequency-based EpiLink-like option; the EM option crashed. When SSN was unavailable, the results were: Splink 579,000 TP (3 FP), fastLink 582,000 TP (137 FP), and Match*Pro 442,000 TP (3 FP); the EM option had 516,000 TP (442 FP). The fastLink developers are working on adding Active Learning.

## Conclusion

Overall, Splink and fastLink performed better than Match*Pro. A likely explanation is that only Splink and fastLink provide match probability and are open source. Splink (Python) and fastLink (R) require basic user programming skills. The results will be updated once the final report has been approved.

